# Supplementary material for: In Vitro Techniques Using the DaisyII Incubator for the Assessment of Digestibility: A Review
Source: Animals (Basel). 2020 Apr 29;10(5):775. doi: 10.3390/ani10050775 (PMC7278437; doi:10.3390/ani10050775)
Supplement: Supplementary file 1 [file animals-10-00775-s001.pdf]

animals

MDPI

Review

Supplementary File: In Vitro Techniques Using the Daisy^{II} Incubator for the Assessment of Digestibility: A Review

Sonia Tassone [1,*], Riccardo Fortina [1] and Pier Giorgio Peiretti [2]

Table S1. Practical recommendations on the use of the Ankom Daisy^{II} incubator (AD^{II}).

		Step	Further Researches
1	**Sample preparation**		
1a	Pre-rinse F57 filter bags in acetone for 3–5 ′ and completely air-dry		
2	Weight samples; 0.250 g (or 0.500) ±0.05, in triplicate	1. Number the bags with a solvent resistant marker<br>2. Homogenized sample<br>3. Weight directly into filter bag<br>4. Clean the top edge of the bag sealing area to avoid sample particles	Test F58 filter bags for AD^{II} [28]
2a	Seal the bags with heat sealer; set 4–5 for 2–3 ″	5. Seale no more than 4 mm from the open end of F57	
2b	Place bags in the jar with a blank bag (up to 25 samples)	6. Distribute bags on both sides of the perforated agitator baffle<br>7. Place the same sample in three different jars to reduce influence of position of the jar in the incubator<br>8. Use a standard in each jar	Study the effects of jar position in AD^{II}
3	Prepare and pour the buffer solutions in the digestion jars	9. Do not store the buffer for further analysis<br>10. Adjuste pH at 6.8 (at 39 °C)	Define a buffer solution to compare experiments
4	Turn on heat and agitation switches	11. Allow temperature of digestion jars to equilibrate for at least 20–30 ′	Modify AD^{II} to allow the same speed of rotation of all jars
5	Rumen fluid collection	12. Use donor animals with feed-controlled diets<br>13. Maintain anaerobic conditions and temperature (39 °C)<br>14. Filter rumen fluid with cheese clothes	Define a standard protocol to collect, transport and store the inoculum.
6	Add 400 mL rumen fluid to each jar	15. Purge the jar with CO_2 for 30 ″	
7	Incubate 48 h	16. Check the rotation of jars and the temperature (39 °C)	
8	Remove jars and drain fluid	17. Rinse bags with cold tap water until water is clear	Standardize the washing of bags (type and time)
9	Oven dry and weight samples	18. Store the bags in a freezer for postponed analysis	

	Formulae	
10a	$TDMD_{AD}$II/FM)	%: 100 − (W_4 − (W_1 × C_1)) × 100 / W_2
10b	$TDMD_{AD}$II/DM	%: 100 − (W_4 − (W_1 × C_1)) × 100 / (W_2 × DM g/g) or 100 − ((100 − NDFD) × (NDF / 100))
		g/g: 1 − (W_4 − (W_1 × C_1)) × 1000 / (W_2 × DM g/Kg)
		g/kg: 1000 − (W_4 − (W_1 × C_1)) × 1000 / (W_2 × DM g/g)
10c	$ADMD_{AD}$II/FM	%: 100 − (W_3 − (W_1 × C_1)) × 100 / W_2
11d	NDFD/NDF	%: (NDF_{OM} *ai.* − NDF_{OM} *pi.*) × 100 / NDF *ai.*
		%: 100 − (W_3 − (W_1 × C_1)) × 100 / (W_2 × (NDF *ai.* g/g FM))
		g/g: 1 − (W_3 − (W_1 × C_1)) × 1000 / (W_2 × NDF *ai.* g/kg FM)
		g/kg: 1000 − (W_3 − (W_1 × C_1)) × 1000 / (W_2 × NDF *ai.* g/g FM)
		1 − ((100 − TDMD) / NDF%DM *ai.*)) × 100
11e	dNDF/DM	%: % NDF%DM *ai.* × (NDFD / 100)

AD^{II} = Ankom Daisy^{II} Incubator; $ADMD_{AD}$^{II} = apparent dry matter digestibility measured with AD^{II}; dNDF = digestible neutral detergent fiber; FM= fresh matter; NDFD = neutral detergent fiber digestibility; $TDMD_{AD}$^{II} = true dry matter digestibility measured with AD^{II}; TDMD = true dry matter digestibility. W_1: F57 weight; W_2: sample weight (without the bag) *ante*-incubation; W_3: sample weight (with the bag) *post*-incubation; W_4: sample weight (with the bag) NDF treatment; C1: F57 *ante/post* incubation (final oven-dried weight/original blank bag weight); *ai.*: *ante-* incubation; *pi.*: *post-* incubation.

Table S2. Variability (CV, %) of the Ankom Daisy^{II} incubator (AD^{II}) after 48 h of incubation.

Variability	ADMD	TDMD	NDFD	AOMD	TOMD	Ref.
Instrument	3.6	4.1	14.6	6.2	4.5	[23]
			2.8			[89]
			9.1			[90]
Within laboratory			6.8			[91]
Between laboratory			10.5			[91]
Within run		14.8	23.6			[87]
Between run		4.7	12.4			[87]
Between jars		2.6				[72]
Within sample		<1–3	2–4.5			[21]
		3.5				[72]

ADMD = apparent dry matter digestibility; TDMD = true dry matter digestibility; NDFD = neutral detergent fiber digestibility; AOMD = apparent organic matter digestibility; TOMD = true organic matter digestibility.

Table S3. Linear equation between Ankom Daisy^{II} incubator (AD^{II}) at 48 h and other digestibility systems.

Instrument	In vivo (vv)	In situ (is)	Tilley and Terry (TT)	Samples	Ref.
**AD^{II}**	$ADMD_{vv} = 0.335 + 0.402 \times TDMD_{AD^{II}}$ ($r^2 = 0.85\%$, n = 24, $p < 0.001$)	$TDMD_{is} =0.27 + 0.904 \times TDMD_{AD^{II}}$ ($r^2 = 0.81\%$, n = 115, $p < 0.001$)	$ADMD_{TT} = 0.101 + 0.641 \times TDMD_{AD^{II}}$ ($r^2 = 0.63\%$, n = 115, $p < 0.001$)	Meadow hay, fine fescue straw	[87]
			$ADMD_{TT} =8.88 + 0.84 \times ADMD_{AD^{II}}$ ($r_2 = 0.81$, n = 17 SD = ±6.24)	Forages, concentrates, protein supplements	[73]
		$NDFD_{is} = 6.39 + 0.74 \times NDFD_{AD^{II}}$ ($r_2 = 0.94$, n = 18, SD = ±1.96)		Hays	[93]
		$NDFD_{is} = 7.31 + 0.95 \times NDFD_{AD^{II}}$ ($r_2 = 0.98$)		Hays	[89]

ADMD = apparent dry matter digestibility; TDMD = true dry matter digestibility; NDFD = neutral detergent fiber digestibility.

Table S4. Acronyms for digestibility trials.

Parameter	Acronyms		Measured
Apparent dry matter digestibility	ADMD	$ADMD_{vv}$	In vivo
		$ADMD_{vt}$	In vitro
		$ADMD_{AD^{II}}$	AD^{II} Incubator procedure
		$ADMD_{TT}$	TT procedure
True dry matter digestibility	TDMD	$TDMD_{is}$	In situ
		$TDMD_{vt}$	In vitro
		$TDMD_{AD^{II}}$	AD^{II} Incubator procedure
Neutral detergent fiber digestibility (% NDF)	NDFD	$NDFD_{vv}$	In vivo
		$NDFD_{is}$	In situ
		$NDFD_{vt}$	In vitro
		$NDFD_{AD^{II}}$	AD^{II} Incubator procedure
		$NDFD_{TT}$	TT procedure
Digestible NDF (% DM)	dNDF	$dNDF_{is}$	In situ
		$dNDF_{vt}$	In vitro
		$dNDF_{AD^{II}}$	AD^{II} Incubator procedure
Apparent organic matter digestibility	AOMD	$AOMD_{vv}$	In vivo
		$AOMD_{is}$	In situ
		$AOMD_{vt}$	In vitro
		$AOMD_{AD^{II}}$	AD^{II} Incubator procedure
True organic matter digestibility	TOMD	$TOMD_{vv}$	In vivo
		$TOMD_{is}$	In situ
		$TOMD_{vt}$	In vitro
		$TOMD_{AD^{II}}$	AD^{II} Incubator procedure

© 2020 by the authors. Licensee MDPI, Basel, Switzerland. This article is an open access article distributed under the terms and conditions of the Creative Commons Attribution (CC BY) license (http://creativecommons.org/licenses/by/4.0/).
